# Classification of racehorse limb radiographs using deep convolutional neural networks

**DOI:** 10.1002/vro2.55

**Published:** 2023-01-29

**Authors:** Raniere Gaia Costa da Silva, Ambika Prasad Mishra, Christopher Michael Riggs, Michael Doube

**Affiliations:** ^1^ Department of Infectious Diseases and Public Health City University of Hong Kong Hong Kong SAR China; ^2^ Department of Veterinary Clinical Services Hong Kong Jockey Club Hong Kong SAR China

**Keywords:** deep learning, machine learning, radiograph, Thoroughbred racehorse

## Abstract

**Purpose:**

To assess the capability of deep convolutional neural networks to classify anatomical location and projection from a series of 48 standard views of racehorse limbs.

**Materials and methods:**

Radiographs (*N* = 9504) of horse limbs from image sets made for veterinary inspections by 10 independent veterinary clinics were used to train, validate and test (116, 40 and 42 radiographs, respectively) six deep learning architectures available as part of the open source machine learning framework PyTorch. The deep learning architectures with the best top‐1 accuracy had the batch size further investigated.

**Results:**

Top‐1 accuracy of six deep learning architectures ranged from 0.737 to 0.841. Top‐1 accuracy of the best deep learning architecture (ResNet‐34) ranged from 0.809 to 0.878, depending on batch size. ResNet‐34 (batch size = 8) achieved the highest top‐1 accuracy (0.878) and the majority (91.8%) of misclassification was due to laterality error. Class activation maps indicated that joint morphology, not side markers or other non‐anatomical image regions, drove the model decision.

**Conclusions:**

Deep convolutional neural networks can classify equine pre‐import radiographs into the 48 standard views including moderate discrimination of laterality, independent of side marker presence.

## INTRODUCTION

Deep convolutional neural networks (DCNN) are a class of machine learning (ML) methods with the potential to augment radiological diagnosis and research.^1^, ^2^ ML has been used in human^3^, ^4^, ^5^, ^6^ and veterinary^7^, ^8^, ^9^, ^10^ medicine for a few decades. Until the early 2010s, application of ML to radiographs required experts to define a set of features (called radiomics^11^, ^12^); the features were used to create models using methods like linear regression, decision trees and support vector machines. DCNN differs from previously mentioned ML because features are created in a process called convolution, which aggregates information of each pixel and its local neighbours across the entire image and is refined during training without the input of experts. So far, most DCNN studies are based on human patients.^13^ Humans have a limited range of morphological variation, while centralisation of radiographic services in large hospitals leads to imaging datasets that are consistent and numerous enough to be used in the development of DCNN.^14^ Veterinary species are typically radiographed in decentralised small practices, with a relatively much lower case‐load than human radiographic practices, with highly variable ranges of views and examination conditions. Within‐species variations may be large such as in dogs, which have a tremendous range of sizes and morphologies. As such, obtaining sufficient numbers and consistency of images is difficult and applying DCNN in veterinary radiology presents a diverse set of challenges.

In such a scenario, Thoroughbred racehorses in Hong Kong are a useful population, because they are centrally managed by the Hong Kong Jockey Club (around 1200 horses at any one time). This single breed of fit, young, nearly all castrated males represents an unusually homogeneous and large study population that is under continuous veterinary supervision. All Hong Kong racehorses must undergo a pre‐import veterinary examination that includes radiography of the limbs to screen for orthopaedic anomalies.^15^ A standard set of 48 radiographs (Table S1) is reviewed to assess the horse's suitability for racing before importation to Hong Kong. Given the high values of these animals, and the financial and welfare costs of later career‐disrupting injury, radiographs are taken to a high standard of consistency and given close scrutiny. Radiograph sets are stored in a specialized database, *Asteris Keystone*. To the best of the authors’ knowledge, there are no existing DCNN tools in use for in vivo Thoroughbred racehorses or for equine radiology generally.

Our objective was to establish an end‐to‐end DCNN‐based diagnostic assistant for the analysis of the 48‐image pre‐import radiographic series used in the Hong Kong Jockey Club protocol. In this initial study, we aimed to determine the most promising DCNN architectures for our application without looking at optimal hyperparameters. We investigated the accuracy of current open source and freely available DCNN architectures to classify equine pre‐import radiographs into the 48 standard views, including left–right and forelimb–hindlimb discrimination. Limb anatomy distal to the carpus (wrist; colloquially ‘knee’) and tarsus (ankle; colloquially ‘hock’) is nearly identical between forelimbs and hindlimbs, with only the third digit present and vestigial second and fourth metapodials.^16^ The metacarpophalangeal and metatarsophalangeal joints (fetlocks) are examined in detail due to their frequent involvement in career‐limiting injury.^17^

## MATERIALS AND METHODS

### Study design

The Hong Kong Jockey Club retrospectively collected and provided pre‐import radiographic sets. The authors selected complete sets to use and no further selection criteria were applied. Given the nature of anonymised, retrospective studies being conducted in this project, our institutional ethics committee determined that their approval was not required.

### Datasets

**FIGURE 1:**
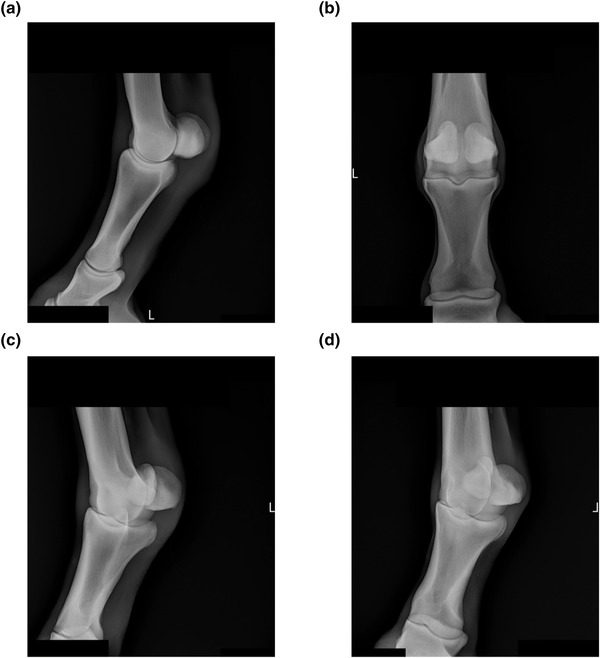
Example of radiographs after metadata text burned into the image pixels was redacted with black rectangles (see corners of the radiographs). (a) Left Fore Fetlock LM. (b) Left Fore Fetlock DP. (c) Left Fore Fetlock DLPMO. (d) Left Fore Fetlock DMPLO. Note the similar appearance of the two oblique views (c and d)

The Hong Kong Jockey Club provided anonymised radiographic image sets from 212 racehorses that had been submitted for pre‐import examination (10,681 radiographs in total). Each set was made by one of 10 veterinary clinics outside of Hong Kong that used their own imaging devices and databases. Each set comprised 48 or more radiographs of the appendicular skeleton in DICOM format with acquisition modality attribute value of CR or DX.^15^ Anonymisation removed details of acquisition hardware used by the veterinary clinics. We extracted metadata detailing the anatomical region and projection (‘view’) from the DICOM headers (using the pydicom library [version 2.2.2]; https://pydicom.github.io/) or from text burned in the image pixels (manually retyping using Label Studio; https://labelstud.io/) and stored it in a CSV file. Burned‐in text was redacted using ImageJ (version 1.53d; https://imagej.net/software/imagej/) by placing a rectangular region of interest over the text area and filling with black (0) pixels (Figure 1).

**FIGURE 2:**
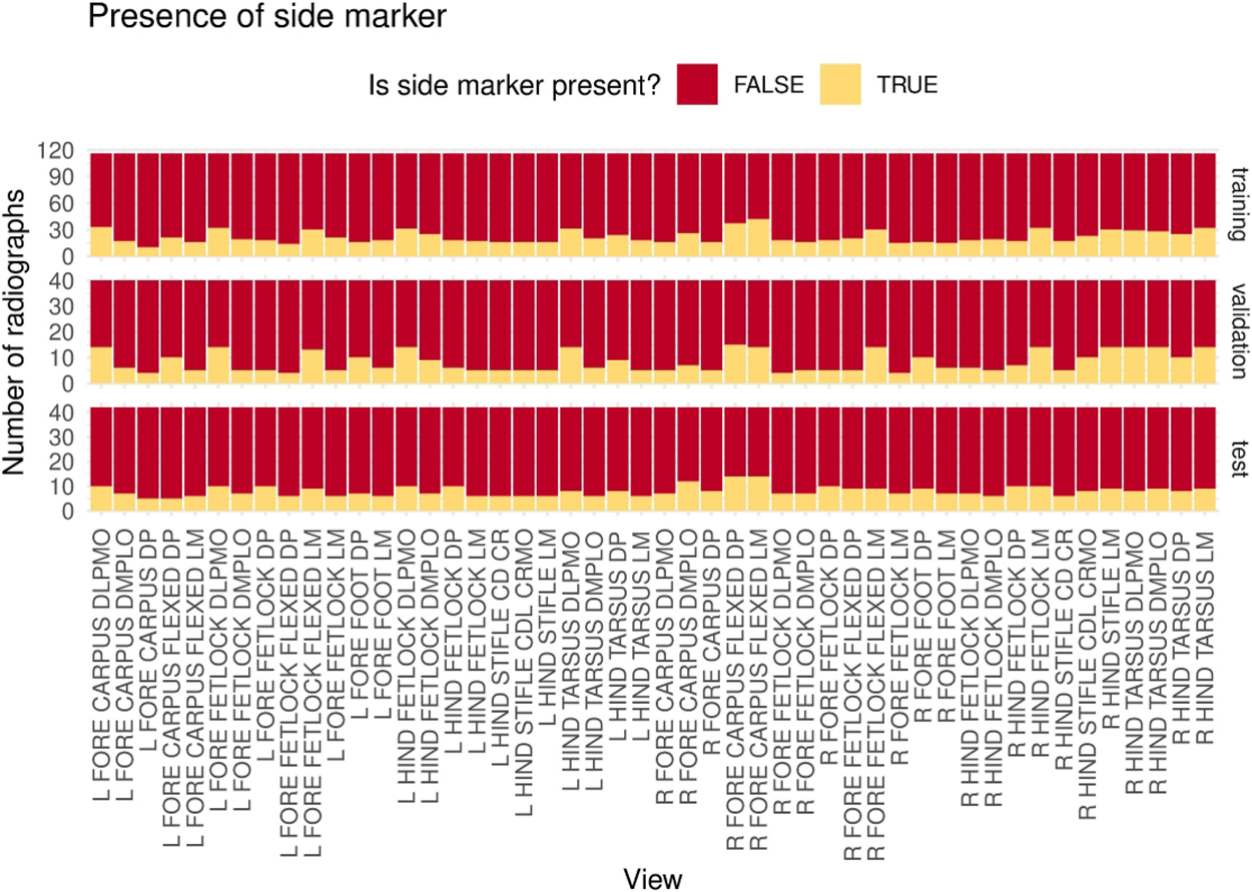
Distribution of radiographs with side marker (left of right limb) among all views within the training, validation and test image sets

View information was processed using Python (version 3.9.9; https://www.python.org/) and Pandas (version 1.3.5; https://pandas.pydata.org/) to standardise the view names. The view information of each individual radiograph was manually reviewed by the first author comparing it to the image. Radiographs with incorrect view information had their view information corrected (30 radiographs) and additional or duplicated radiographs in a set were marked for exclusion in the next step (180 radiographs). Apart from random human error, the view information was assumed to be correct and therefore the authors only investigated DCNN's errors in the classification. Python and Pandas were used to verify if the set was complete, taking manual corrections into consideration. A set was complete only if it contained exactly one of all 48 specified views, leading to a perfectly balanced dataset. The Python script reported 198 sets as complete and 14 sets as incomplete. The 198 complete sets were split using Python and scikit‐learn (version 1.0.2; https://scikit‐learn.org/stable/) into training (116), validation (40) and test (42) sets. Only 19.3% of the radiographs had a side marker indicating laterality (left or right limb) (Figure 2). Incomplete sets were excluded from further analysis.

### Data preparation

**FIGURE 3:**
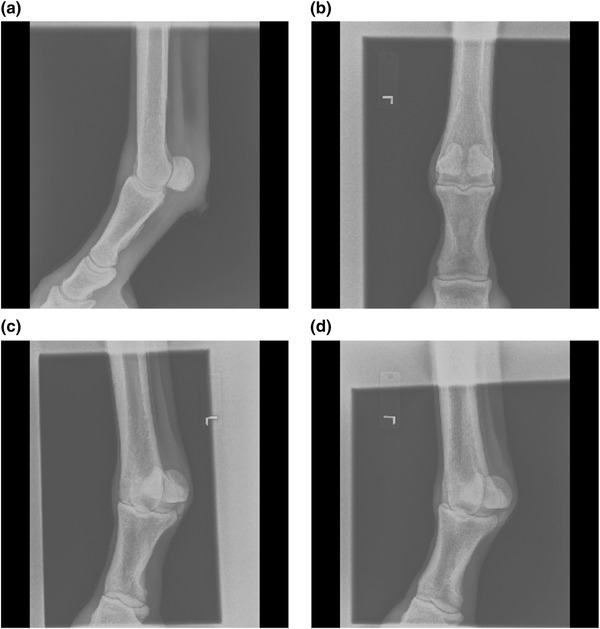
Example of radiographs after pre‐processing into 250 × 250 pixel 16‐bit single‐channel image. (a) Left Fore Fetlock LM. (b) Left Fore Fetlock DP. (c) Left Fore Fetlock DLPMO. (d) Left Fore Fetlock DMPLO. Note the similar appearance of the two oblique views (c and d). An extended gallery of images is available at Figshare (https://doi.org/10.6084/m9.figshare.c.5921813)

The radiographs of the 198 complete sets were rotated by 90° increments and/or mirrored into the standard anatomical viewing orientation (head of the horse in the top left corner of the image and lateral side of the horse in the left side of the image), centred on a square black (pixel value 0) canvas fit to the radiograph's long axis, downsampled to 250 × 250 pixels by nearest‐neighbour interpolation from the input image; then stored in 16‐bit Tag Image File Format (TIFF) with a greyscale look‐up table stretched to display 0 as black and the radiograph's maximum pixel value as white (Figure 3).

### Deep convolutional neural network architecture

Based on previous DCNN architectures,^18^, ^19^ we adapted six deep learning architectures (Table 1), available as part of the open source machine learning framework PyTorch,^20^ to output a vector with 48 values equal to the probability of the input image belonging to each of the 48 standard radiographic views. In our implementation, the 250 × 250‐pixel single‐channel image was augmented using random zoom, random spatial crop, random histogram shift and scaled down to 224 × 224 pixels. We trained each of the selected deep learning architectures without transfer learning (pre‐trained weights were not available for single channel images) for 128 epochs with batch size of 32 images, using SGDM as the optimiser. The best DCNN architecture was also trained with different batch sizes 8, 16, 32 and 48. Computation was performed on a Precision 5820 workstation (Dell Hong Kong) equipped with an Intel(R) Xeon(R) W‐2123 3.60 GHz CPU, 64 GiB (4 × 16 GiB DDR4 2666 MHz) of RAM and NVIDIA Quadro RTX 4000 GPU running Ubuntu 21.10 (Linux 5.13; https://ubuntu.com/), Python, NumPy (version 1.21.2; https://numpy.org/), PyTorch (version 1.10.2; https://pytorch.org/) and CUDA (version 11.3; https://docs.nvidia.com/cuda/archive/11.3.0/).

**TABLE 1:** Deep learning models used for classification

**Architecture**	**Number of parameters**	**Relative number of parameters**
DenseNet‐121	7978856	0.29
Inception V3	27161264	1.00
MobileNet V3	5483032	0.20
ResNet‐18	11689512	0.43
ResNet‐34	21797672	0.80
ResNet‐50	25557032	0.94

### Statistical analysis

All statistical analysis was conducted on the Precision 5820 workstation (Dell Hong Kong) described previously.

During model training, each DCNN architecture had, at the end of each epoch, loss, top‐1 accuracy and multiclass area under the receiver operating characteristic curve (ROC AUC) with the strategies one‐versus‐one and unweighted mean calculated for the training and validation set (116 and 40 radiographs, respectively) using scikit‐learn with Python and NumPy; then stored in Comet (https://www.comet.ml) for further analysis.

After model training, we selected the model with the highest top‐1 accuracy for each DCNN architecture and ran it on the test set (42 sets of 48 views). Top‐1 accuracy and AUC were calculated using scikit‐learn with Python and NumPy; then stored in Comet for further analysis.

The DCNN architecture with the highest top‐1 accuracy on the test set was further analysed. Descriptive statistics and tests for correlation (Pearson's chi‐square test) were conducted using R version (implemented in R: a language and environment for statistical computing, R Foundation for Statistical Computing, Vienna, Austria, 2015 [R version 4.2.1]; https://www.R‐project.org) (chisq.test function for Pearson's chi‐square test).

Further details of the protocol for statistical analysis are available at figshare https://doi.org/10.6084/m9.figshare.20962264

### Class activation mapping

To gain insight into the features used by the DCNN architecture with the highest top‐1 accuracy to determine the view classification probabilities, a class activation map was generated for each radiograph in the test set using Monai (version 0.8.0; https://monai.io/) and matplotlib (version 3.5.1; https://matplotlib.org/) with Python, NumPy, PyTorch and Cuda on the Precision 5820 workstation (Dell Hong Kong) described previously.

### Model availability

A copy of the source code and model weights of each of the six deep learning architectures are provided in Tables S2–S4.

## RESULTS

**FIGURE 4:**
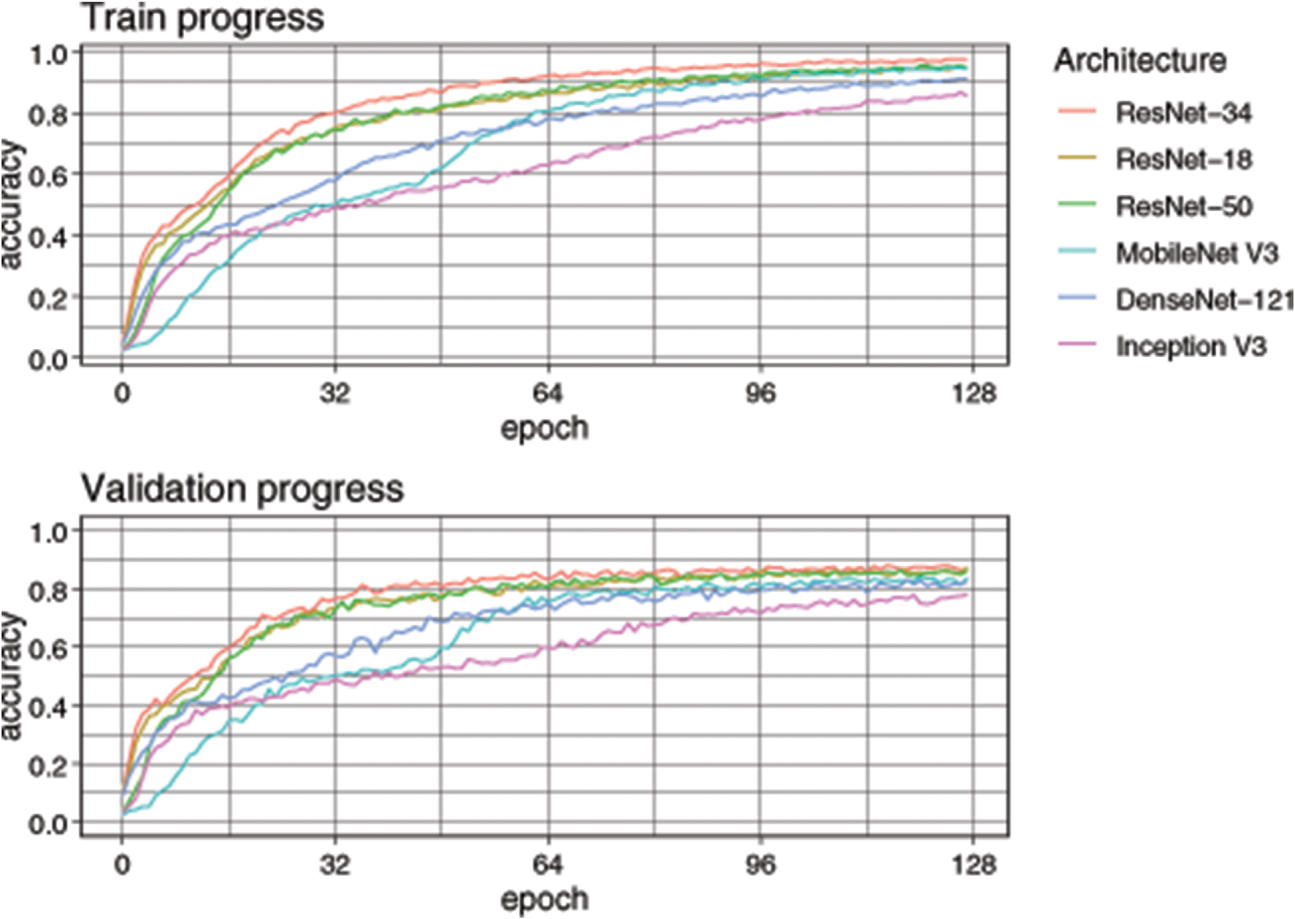
Progress of metrics during training to classify racehorse body part including view and laterality from radiographs. An epoch is one pass over all images

**FIGURE 5:**
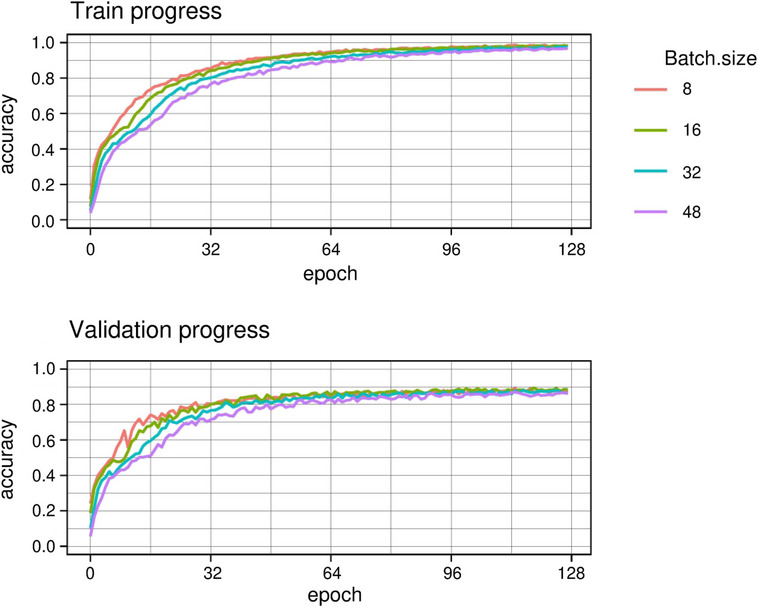
Progress of metrics during training to classify racehorse body part including view and laterality from radiographs using ResNet‐34 architecture and different batch size. An epoch is one pass over all images

The DCNN architecture with the highest top‐1 accuracy 0.841 in the test set was ResNet‐34, followed by ResNet‐18 with a slightly lower top‐1 accuracy (0.832) (Table 2). Increased model size did not result in higher accuracy, with ResNet‐50 achieving accuracy of only 0.819. ResNet‐34 achieved higher top‐1 accuracy at earlier epochs than the other models during training and validation (Figure 4). MobileNet V3 had a low accuracy at the beginning of the training, but finished with accuracy above 0.8 (Figure 4) despite being the smallest architecture (Table 1). ResNet‐34 was retained for further analysis and the other models discarded. ResNet‐34 achieved its highest top‐1 accuracy (0.878) with a batch size of eight (Table 3). A batch size of eight achieved higher top‐1 accuracy at earlier epochs than other batch sizes during training and validation, while top‐1 accuracy converged in the last quarter of epochs (Figure 5).

**TABLE 2:** Architectures, sorted by accuracy, for the classification of racehorse body part including view and laterality from radiographs and their respective metrics with batch size 32

**Architecture**	**Accuracy**	**ROC AUC**
ResNet‐34	0.841	0.998
ResNet‐18	0.832	0.998
ResNet‐50	0.819	0.998
MobileNet V3	0.810	0.997
DenseNet‐121	0.792	0.997
Inception V3	0.737	0.996

**TABLE 3:** Metrics for different values of batch size for ResNet‐34

**Batch size**	**Accuracy**	**ROC AUC**
8	0.878	0.998
16	0.858	0.998
32	0.841	0.998
48	0.809	0.997

**FIGURE 6:**
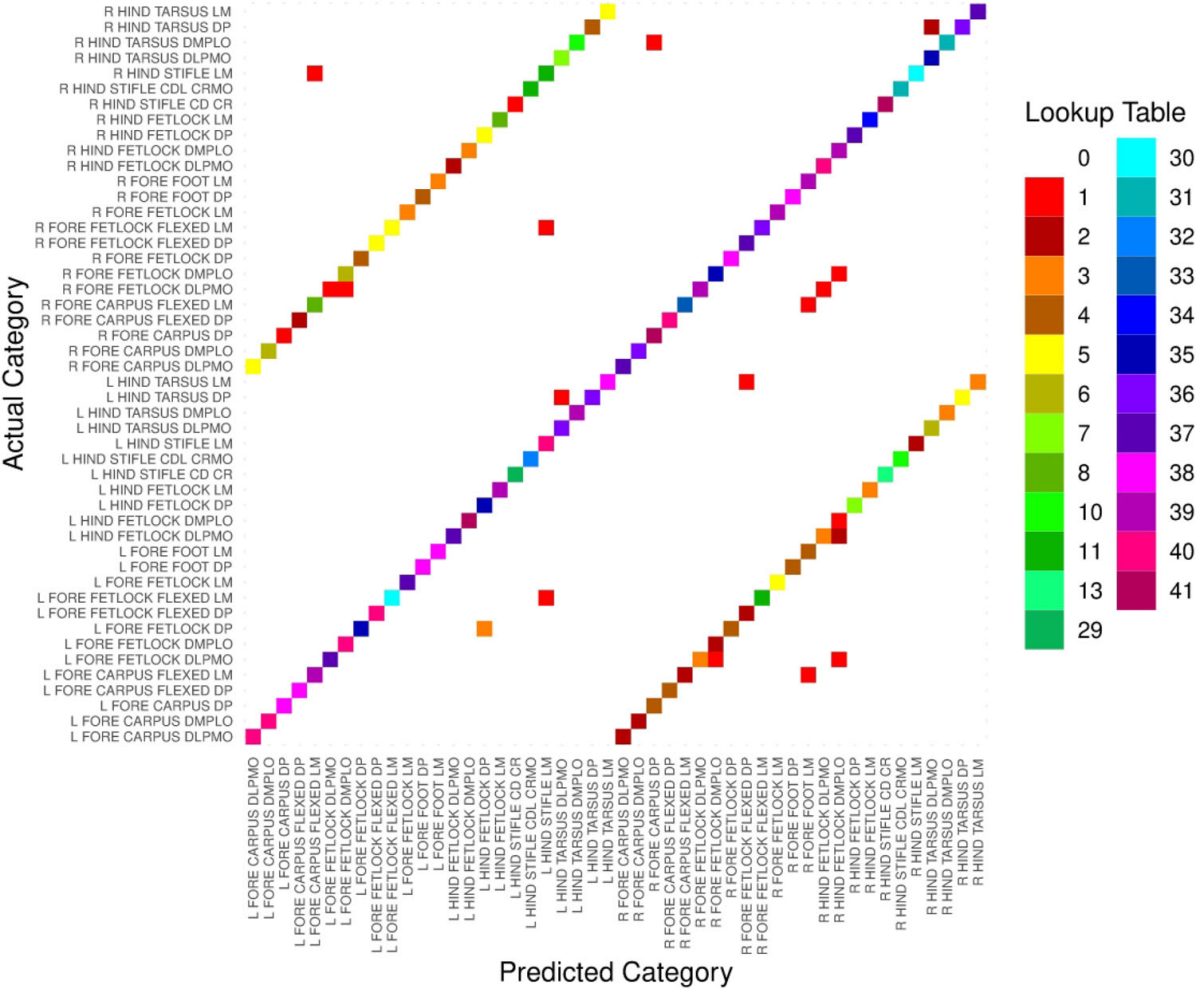
Confusion matrix for classification of racehorse body part including view and laterality from radiographs by the best performing architecture, ResNet‐34 (batch size = 8). The central diagonal line indicates a high degree of correct classification, while the two shorter parallel diagonal lines indicate predictions where only left or right laterality was misclassified but the view and fore–hind designation was correct.

For ResNet‐34 (batch size = 8), the most common misclassification (91.8% of cases) involved assignment of incorrect laterality (left–right disagreement) to an otherwise correctly identified view (Figure 6). A side marker was present in 19.0% of the radiographs used for testing and 18.2% of correctly classified radiographs used for testing. Side markers appeared with greater frequency in misclassified radiographs and, on average, side marker presence had a significant negative correlation with correct classification (Pearson's *Χ* ^2^ [1, *N* = 2016] = 6.31, *p* = 0.012, *r* = 0.0559). Correlation between correct classification of the radiograph and side marker presence varied by view (see Table S5) because the number of radiographs containing a side marker was different for each view in the training set (Figure 2).

**FIGURE 7:**
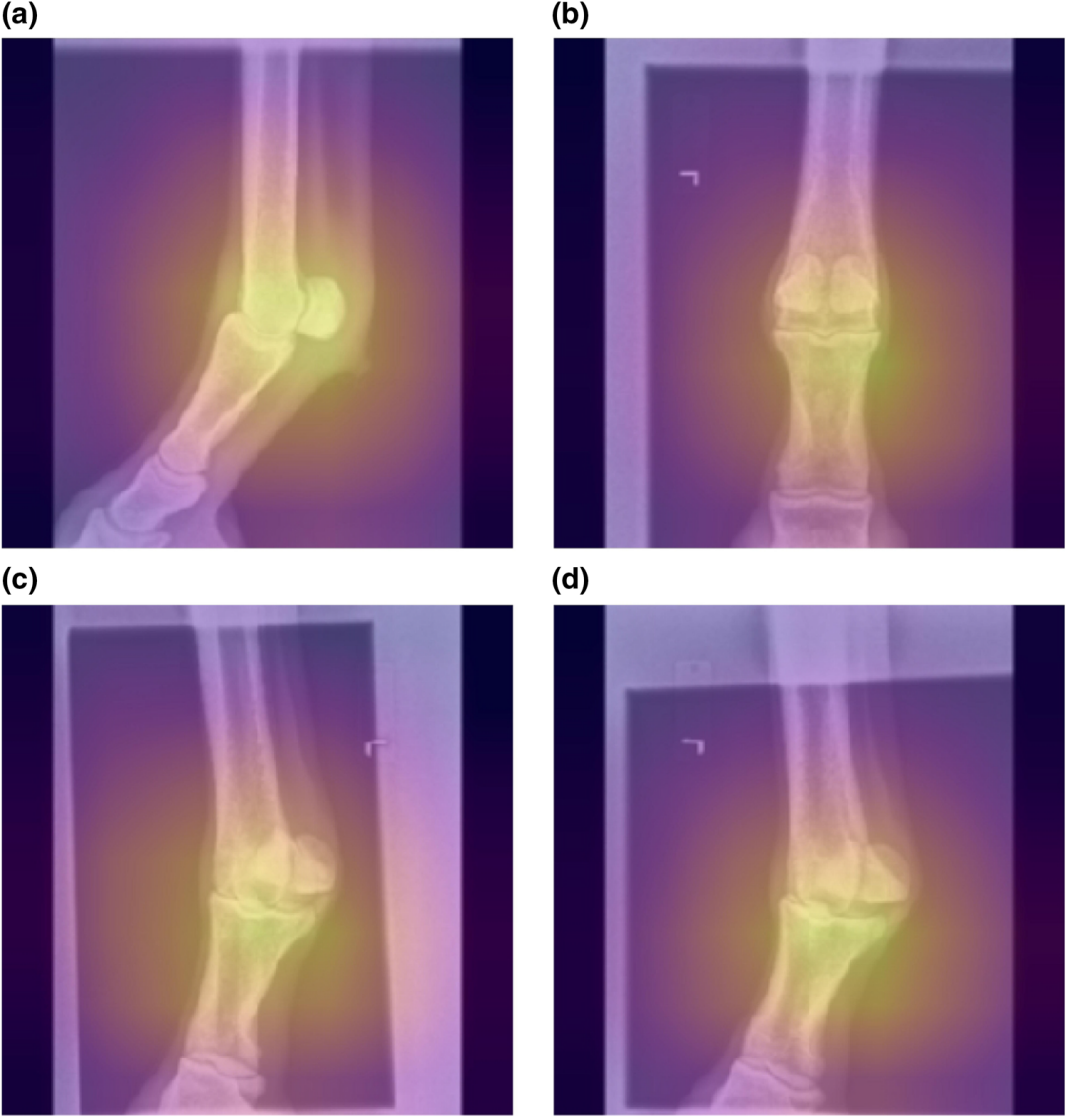
Class activation maps applied as overlays to 224 × 224 pixel radiographs. Yellow indicates higher contribution and purple a lower contribution, of the image region to the classification. Note that the ‘L’ side marker is not highlighted by the class activation map, indicating that it did not contribute to the model weighting, while the metacarpophalangeal joint (fetlock) is brightly identified. (a) Left Fore Fetlock LM. (b) Left Fore Fetlock DP. (c) Left Fore Fetlock DLPMO. (d) Left Fore Fetlock DMPLO. An extended gallery is available at Figshare (https://doi.org/10.6084/m9.figshare.c.5921816).

Class activation maps^21^ indicated that anatomical features, in particular joint morphology, were the primary source of signal used by the model to determine view classification and that side markers, when present, were not strongly weighted by the model (Figure 7).

Redaction of burned‐in text was performed in 26.2% of the radiographs in the test group and in 23.8% of the correctly classified radiographs used for testing. Redaction frequency in the two sets was significantly different (Pearson's *Χ* ^2^ [1, *N* = 2016] = 42.1, *p* ⩽ 0.01, *r* = 0.144). Redaction was concentrated in misclassified radiographs and, on average, redaction had a negative correlation with correct classification.

Performance was not evenly spread across the views (Table S5), with ResNet‐34 achieving greater than 0.95 best top‐1 accuracy (40 or more correct out of 42) for 10 views, but achieving less than 0.75 best top‐1 accuracy (31 or fewer correct out of 42) for five views. No projection achieved more than 95% best top‐1 accuracy for both lateralities (left and right). Stifle caudocranial and stifle lateromedial projections had one laterality with top‐1 accuracy above 0.95 and the other laterality with top‐1 accuracy below 0.75.

Ignoring left–right laterality classification, our model reached an accuracy of 99.0%, which included the discrimination of very similar examination views, for example, fore fetlock DLPMO and fore fetlock DMPLO (Figure 3).

## DISCUSSION

Here, we have shown that deep learning architectures, available in the PyTorch open source machine learning framework,^20^ can classify radiographic views (anatomical location and projection) of horse limbs with remarkable accuracy despite the close similarity among many of the views and heavily downsampled resolution (224 × 224 pixels) used for training, validation and testing. To the best of the authors’ knowledge, our study is the first to apply deep learning for equine limb radiographs, joining others who have investigated the application of deep learning architectures to human limb radiographs.^22^, ^23^

We defined 48 classes to fully describe the radiographs, including exam body part, exam view and laterality, which is up to six times more classes than studies in humans.^22^, ^23^ The high number of classes implies that there is a small likelihood (2.1%) of an image's view being correctly classified by chance; however, our best model was correct 87.8% of the time. Accuracy is smaller than that reported in studies in humans^22^, ^23^ and must be interpreted taking into consideration the differences between the data used in each of the studies; for example, the presence of side markers (only 19.3% of our images contain side markers vs. 99% of the radiographs used in studies in humans^22^, ^23^) and redaction of radiographs to remove text burned in the image pixels (28.3% of our images were redacted vs. none of the radiographs used in studies in humans^22^, ^23^).

The most common misclassification of our model involved assignment of a correct view to the incorrect laterality. In a human study, correct classification of laterality was strongly correlated with the side marker, as two‐thirds of the misclassification was with radiographs without a side markers.^22^ Another human study found that side markers may be an important classification feature based on class activation maps.^23^ In contrast, we observed a negative correlation between side marker presence and correct classification, while class activation maps indicated little contribution from side markers.

Random guessing of left and right should result in 50% correctly assigned (assuming no other misclassifications), however, our best model detected side 88.4% correctly, implying that 38.4 times out of the remaining 50 times the model improved on chance based on anatomical information alone. It is somewhat surprising that laterality detection was better than chance, however, asymmetry of limb bone dimensions is recognized in humans^24^ and *Equidae*,^25^ which our model appears to give weight to in its radiographic view classification. Horses worldwide are typically handled from their left (‘near’) side and many racehorses do fast track work in one direction only (clockwise in Hong Kong), but limb asymmetry also exists in horses not performing single‐direction training.^25^, ^26^ Previous studies have reported significant differences between right and left limbs; for example, difference in length between right and left third metacarpal bone from Thoroughbred racehorses in Victoria and South Australia^27^; five postmortem measurements were larger in the left femur compared to the right femur from Thoroughbred racehorses in New South Wales, Australia.^28^ Images were supplied individually in random order and not as single‐animal sets or left–right pairs, so direct within‐individual comparisons were not available to the model to use in laterality discrimination. While size may be a contributing factor, yet others such as length–width aspect ratio, cortical thickness or opacity, or trabecular bone texture might also contribute to the model weights. Individual horses commonly have bone dimensions that are asymmetric in the opposite direction to mean population‐level asymmetry,^25^, ^26^ which may help to account for our model's imperfect accuracy in laterality classification.

Radiographer handedness could conceivably affect image properties and the accuracy of our models, however, images were taken with a standardised protocol that should eliminate operator effects.

Redacting radiographs to remove burned‐in text in the pixels (Figure 1) was also positively correlated with misclassification. This was not a surprise because the redaction reduces the amount of bone represented in the radiograph. For example, the redacted radiographs in Figure 1 did not have white pixels in the top margin and this might be a strong feature for the model to classify the radiograph as fore hoof lateromedial. We did not investigate this further because we expect veterinary clinics to move the horse case information from the pixels to DICOM headers in the near future and redaction will no longer be necessary.

Batch size is negatively correlated with top‐1 accuracy. This is because smaller batch sizes increase the chances of new gradient information producing a positive impact on training.^29^

Few studies have focused on classifying radiographs based on the examination view. A study used 5085 radiographs to train a model based on ResNet‐152 that achieved 96% accuracy in the task of classifying 404 human hand radiographs into posteroanterior, lateral or oblique views,^30^ to which our model compares favourably by achieving 99% accuracy over 24 laterality‐neutral views.

A common pitfall of deep learning applications is that models are accurate and precise but not robust^31^ in that the model achieves low accuracy or precision when new data are used as input. This pitfall is also called ‘dreaded data shift’ or ‘model that does not generalise’. We expect that our models will retain high accuracy with data from other sources given that the radiographs used were already from numerous (10) independent veterinary clinics from outside Hong Kong. Given the paucity of similar collections of radiographs publicly available under an open licence, we have not yet been able to investigate the robustness of our models further. We invite readers to test our models and report back their findings.

The biggest challenge to advance the application of deep learning to non‐human skeletal radiographs is the access to a large collection of radiographs. In our study, we used 9504 radiographs split into 48 different classes, which is orders of magnitude smaller than collections of human radiographs used in other studies,^19^, ^22^, ^23^, ^32^ including MURA,^14^ a ‘public’ dataset of musculoskeletal radiographs containing 40,561 images of seven parts of the human upper limb.

In conclusion, we assessed the capacity of popular deep learning architectures to classify racehorse radiographic views, including view and laterality. After testing different values of batch size, the highest accuracy achieved was 0.878. With batch size of 32, accuracy of tested DCNN architectures was between 0.737 and 0.841. The biggest source of misclassification was laterality, although this was detected much better than chance and used anatomy rather than side markers. Ignoring laterality, our best model achieved accuracy of 0.99. Future studies will explore how to reduce the misclassification due to incorrect laterality by including searching for optimal hyperparameters, additional data in the training steps, using larger images as input and using generative adversarial networks.

## AUTHOR CONTRIBUTIONS

Study concepts/study design: Michael Doube and Raniere Gaia Costa da Silva. Data acquisition: Raniere Gaia Costa da Silva. Data analysis/interpretation: all authors. Manuscript drafting: Raniere Gaia Costa da Silva and Michael Doube. Literature research: Raniere Gaia Costa da Silva and Michael Doube. Statistical analysis: Raniere Gaia Costa da Silva. Manuscript revision for important intellectual content, approval of final version of submitted manuscript and manuscript editing: all authors. All the authors agree to ensure any questions related to the work are appropriately resolved.

## CONFLICTS OF INTEREST

The authors declare they have no conflicts of interest.

## ETHICS STATEMENT

Permission to use pre‐import radiograph sets was granted by the Hong Kong Jockey Club ownership agreement.

## Supporting information

Supporting Information

## Data Availability

The data that support the findings of this study are available from the Hong Kong Jockey Club. Restrictions apply to the availability of these data, which were used under license for this study.
