# Supplementary material for: Classification of racehorse limb radiographs using deep convolutional neural networks
Source: Vet Rec Open. 2023 Jan 29;10(1):e55. doi: 10.1002/vro2.55 (PMC9884469; doi:10.1002/vro2.55)
Supplement: Supplementary file 1 — Supporting Information [file VRO2-10-e55-s001.pdf]

Supporting Information

Table S1 Pre-sale Examination Set

**Anatomical Region**	**View (Abbreviation)**	**View**
Carpus	DP	dorsopalmar
Carpus	DLPMO	dorsal 55º lateral to palmaromedial oblique
Carpus	DMPLO	dorsal 75º medial to palmarolateral oblique
Carpus	FLEXED LM	flexed lateromedial
Carpus	FLEXED DP	flexed dorsal 60º proximal dorsodistal oblique
Fore Fetlock	DP	dorsopalmar
Fore Fetlock	DLPMO	dorsal 45º lateral to palmaromedial oblique
Fore Fetlock	DMPLO	dorsal 45º medial to palmarolateral oblique
Fore Fetlock	FLEXED LM	flexed lateromedial
Fore Fetlock	FLEXED DP	flexed dorsal 125º distal to palmaroproximal oblique
Fore Fetlock	LM	lateromedial
Hind Fetlock	DP	dorsoplantar
Hind Fetlock	DLPMO	dorsal 45º lateral to plantaromedial oblique
Hind Fetlock	DMPLO	dorsal 45º medial to plantarolateral oblique
Hind Fetlock	LM	Lateromedial
Tarsus	DP	Dorsoplantar
Tarsus	DLPMO	dorsal 10º lateral to plantaromedial oblique
Tarsus	DMPLO	dorsal 65º medial to plantarolateral oblique
Tarsus	LM	Lateromedial
Stifle	LM	Lateromedial
Stifle	CD CR	Caudocranial
Stifle	CDL CRMO	caudolateral to craniomedial oblique
Fore Hoof	LM	lateromedial
Fore Hoof	DP	dorsal 60º proximal to palmarodistal oblique

Table S2 Model's source code and weights with batch size 32

Architecture	Source Code	Weights
DenseNet-121	10.6084/m9.figshare.19453334	10.6084/m9.figshare.19453307
Inception V3	10.6084/m9.figshare.19453319	10.6084/m9.figshare.19453307
MobileNet V3	10.6084/m9.figshare.19453349	10.6084/m9.figshare.19453307
ResNet-18	10.6084/m9.figshare.19453274	10.6084/m9.figshare.19453277
ResNet-34	10.6084/m9.figshare.19453289	10.6084/m9.figshare.19453292
ResNet-50	10.6084/m9.figshare.19453304	10.6084/m9.figshare.19453307

Table S3 Model's weights for ResNet-34 with different batch size 32

Batch Size	Weights
8	10.6084/m9.figshare.21673646
16	10.6084/m9.figshare.21673634
32	10.6084/m9.figshare.19453292
48	10.6084/m9.figshare.21673622

Table S4 Model Report Location

Architecture	Jupyter Notebook	Report
DenseNet-121	10.6084/m9.figshare.19453343	10.6084/m9.figshare.19453346
Inception V3	10.6084/m9.figshare.19453328	10.6084/m9.figshare.19453331
MobileNet V3	10.6084/m9.figshare.19453358	10.6084/m9.figshare.19453361
ResNet-18	10.6084/m9.figshare.19453283	10.6084/m9.figshare.19453286
ResNet-34	10.6084/m9.figshare.19453298	10.6084/m9.figshare.19453301
ResNet-50	10.6084/m9.figshare.19453313	10.6084/m9.figshare.19453316

Side Marker Presence and Classification Success

Our null hypothesis was that side marker presence and classification success are dependent in the set of radiographs with a single label when the classification is provided by ResNet-34. The null hypothesis was tested using Pearson's chi-squared test implemented in R: A Language and Environment for Statistical Computing, R Foundation for Statistical Computing, Vienna, Austria, 2015 (R version 4.2.1) https://www.R-project.org

Table S5 Pearson's chi-squared test of side marker presence and classification success for each label

Label	With side marker (%)	Correctly classified (%)	p-value
L FORE CARPUS DLPMO	23.8	95.2	0.4180
L FORE CARPUS DMPLO	16.7	95.2	0.5170
L FORE CARPUS DP	11.9	90.5	0.4400
L FORE CARPUS FLEXED DP	11.9	90.5	0.3950
L FORE CARPUS FLEXED LM	14.3	92.9	0.4630
L FORE FETLOCK DLPMO	23.8	88.1	0.8310
L FORE FETLOCK DMPLO	16.7	95.2	0.5170
L FORE FETLOCK DP	23.8	83.3	0.5170
L FORE FETLOCK FLEXED DP	14.3	95.2	0.5540
L FORE FETLOCK FLEXED LM	21.4	71.4	0.7210
L FORE FETLOCK LM	14.3	88.1	0.3310
L FORE FOOT DP	16.7	90.5	0.0600
L FORE FOOT LM	14.3	90.5	0.3910
L HIND FETLOCK DLPMO	23.8	88.1	0.0429
L HIND FETLOCK DMPLO	16.7	97.6	0.6510
L HIND FETLOCK DP	23.8	83.3	0.1950
L HIND FETLOCK LM	14.3	92.9	0.3280
L HIND STIFLE CD CR	14.3	69.0	0.0027
L HIND STIFLE CDL CRMO	14.3	76.2	0.6570
L HIND STIFLE LM	14.3	95.2	0.5540
L HIND TARSUS DLPMO	19.0	85.7	0.3360
L HIND TARSUS DMPLO	14.3	92.9	0.3280
L HIND TARSUS DP	19.0	85.7	0.3360
L HIND TARSUS LM	14.3	90.5	0.3910
R FORE CARPUS DLPMO	16.7	88.1	0.8310
R FORE CARPUS DMPLO	28.6	85.7	0.7800
R FORE CARPUS DP	19.0	97.6	0.6230
R FORE CARPUS FLEXED DP	33.3	95.2	0.3060
R FORE CARPUS FLEXED LM	33.3	78.6	0.1110
R FORE FETLOCK DLPMO	16.7	92.9	0.4210

R FORE FETLOCK DMPLO	16.7	83.3	0.8530
R FORE FETLOCK DP	23.8	90.5	0.9530
R FORE FETLOCK FLEXED DP	21.4	88.1	0.9340
R FORE FETLOCK FLEXED LM	21.4	85.7	0.1670
R FORE FETLOCK LM	16.7	92.9	0.4210
R FORE FOOT DP	21.4	90.5	0.8550
R FORE FOOT LM	16.7	92.9	0.4210
R HIND FETLOCK DLPMO	16.7	95.2	0.5170
R HIND FETLOCK DMPLO	14.3	92.9	0.4630
R HIND FETLOCK DP	23.8	88.1	0.8310
R HIND FETLOCK LM	23.8	81.0	0.0043
R HIND STIFLE CD CR	14.3	97.6	0.6790
R HIND STIFLE CDL CRMO	19.0	73.8	0.0887
R HIND STIFLE LM	21.4	71.4	0.2340
R HIND TARSUS DLPMO	19.0	83.3	0.4820
R HIND TARSUS DMPLO	21.4	73.8	0.0238
R HIND TARSUS DP	19.0	85.7	0.8730
R HIND TARSUS LM	21.4	88.1	0.9340
